# *osr1* Maintains Renal Progenitors and Regulates Podocyte Development by Promoting *wnt2ba* via the Antagonism of *hand2*

**DOI:** 10.3390/biomedicines10112868

**Published:** 2022-11-09

**Authors:** Bridgette E. Drummond, Brooke E. Chambers, Hannah M. Wesselman, Shannon Gibson, Liana Arceri, Marisa N. Ulrich, Gary F. Gerlach, Paul T. Kroeger, Ignaty Leshchiner, Wolfram Goessling, Rebecca A. Wingert

**Affiliations:** 1 Department of Biological Sciences, Center for Stem Cells and Regenerative Medicine, Center for Zebrafish Research, University of Notre Dame, Notre Dame, IN 46556, USA; 2 Brigham and Women’s Hospital, Genetics and Gastroenterology Division, Harvard Medical School, Harvard Stem Cell Institute, Boston, MA 02215, USA

**Keywords:** kidney, podocyte, nephron, development, zebrafish, *osr1*, *wnt2ba*, *hand2*

## Abstract

Knowledge about the genetic pathways that control nephron development is essential for better understanding the basis of congenital malformations of the kidney. The transcription factors Osr1 and Hand2 are known to exert antagonistic influences to balance kidney specification. Here, we performed a forward genetic screen to identify nephrogenesis regulators, where whole genome sequencing identified an *osr1* lesion in the novel *oceanside* (*ocn*) mutant. The characterization of the mutant revealed that *osr1* is needed to specify not renal progenitors but rather their maintenance. Additionally, *osr1* promotes the expression of *wnt2ba* in the intermediate mesoderm (IM) and later the podocyte lineage. *wnt2ba* deficiency reduced podocytes, where overexpression of *wnt2ba* was sufficient to rescue podocytes and *osr1* deficiency. Antagonism between *osr1* and *hand2* mediates podocyte development specifically by controlling *wnt2ba* expression. These studies reveal new insights about the roles of Osr1 in promoting renal progenitor survival and lineage choice.

## 1. Introduction

The kidney is the organ that cleanses our blood and initiates the process of waste excretion. The portions of the kidney that make this possible are the nephrons, which are composed of a blood filter, tubule, and collecting duct. The blood filter itself is composed of several cellular features including a capillary bed with a fenestrated endothelium. Octopus-like epithelial cells known as podocytes are situated in opposition to a specialized glomerular basement membrane (GBM) surrounding the capillaries [1]. Filtration is accomplished due to the layered ultrastructure of fenestrated epithelium, GBM, and podocytes, which keeps large bulky particles from entering the tubule [2,3]. Podocytes form elaborate cellular extensions and are connected to adjacent podocytes through cell membrane-based protein interactions that create a specialized barrier known as the slit diaphragm [2]. The slit diaphragm allows small or appropriately charged molecules to pass, which initiates a filtrate product that flows into the tubule and is subsequently augmented by specialized solute transporters arranged in a segmental pattern to create a concentrated waste product [1,4]. Within the nephron tubule, the proximal segments perform the bulk of reabsorption, particularly of organic molecules, while the distal segments fine tune the amount of water within the filtrate [5]. Human kidneys, each composed of around a million nephrons, filter all of the blood in our body almost 30 times daily to produce 1–2 quarts of urine (NIDDK). Damage to the specialized cells of the kidney is nearly permanent, as the human kidney has limited regenerative capacity. Therapeutic interventions that can reverse renal damage for patients with acquired kidney diseases and birth defects are urgently needed [6,7,8]. Expanding our understanding about renal development pathways proffers a valuable avenue for addressing these needs.

Early in embryogenesis, the intermediate mesoderm (IM) gives rise to the earliest form of the kidney, or pronephros. The paraxial mesoderm (PM) and lateral plate mesoderm (LPM) flank the developing IM [9,10]. While functional in lower vertebrates, the pronephros is vestigial in mammals [11]. This structure degenerates to give rise to the mesonephros, which is the terminal kidney in fish and amphibians, but in mammals, it is followed by the metanephros [12]. While humans are born with a static number of nephrons in their metanephros [13,14], teleost fishes such as the zebrafish (*Danio rerio*) continue to form nephrons throughout their lifetime and upon injury [15,16]. 

**Figure 1:**
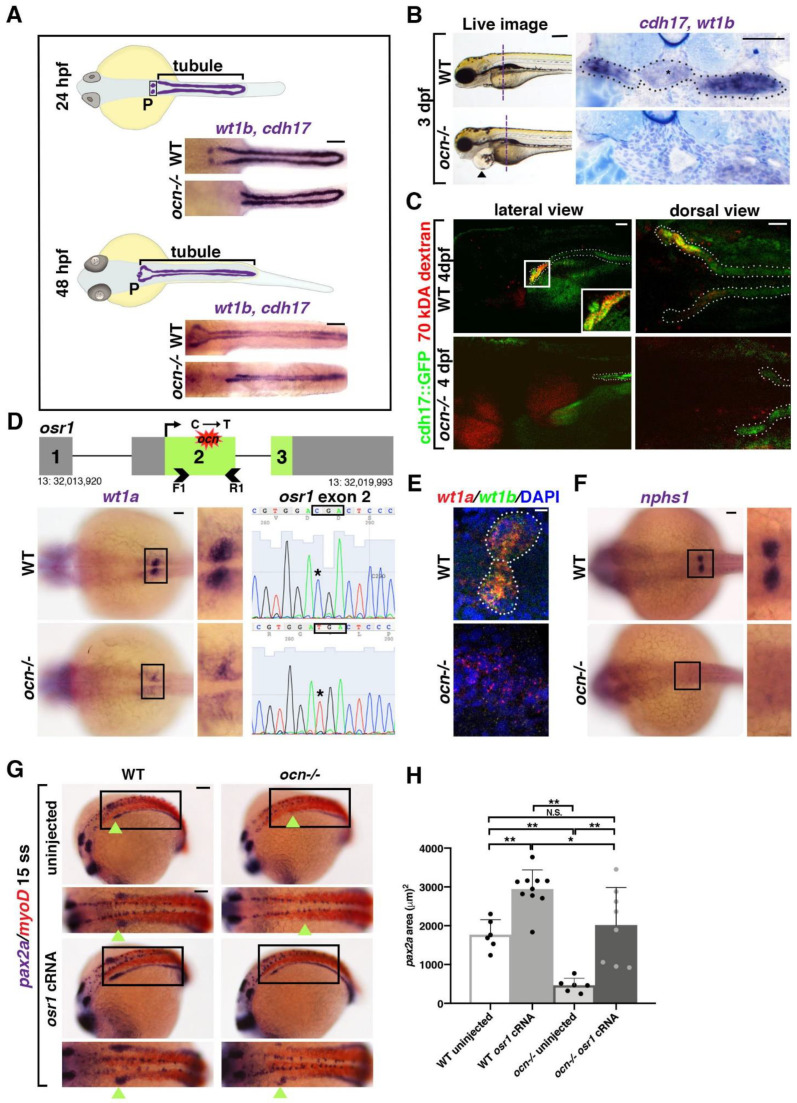
The ENU mutant *ocn* has a proximally abrogated pronephros due to a lesion in the gene *osr1*. (**A**) At 24 hpf, the zebrafish pronephros contains two clusters of podocytes (P) and two nephron tubules. By 48 hpf, the pronephros is functional as the podocyte progenitors have migrated to the midline and fused. In *ocn*−/− mutants, podocyte progenitors (*wt1b*) are reduced at both stages. The pronephric tubules (*cdh17*) were truncated at 24 hpf, which became more dramatic at 48 hpf. Scale bar is 50 μm. (**B**) A live time course of *ocn* revealed pericardial edema beginning at 72 hpf, as indicated by black arrow heads. This fluid imbalance was symptomatic of organ dysfunction. Scale bar is 70 μm. WISH experiments to view podocytes and tubules (*wt1b*, *cdh17*) were also conducted at 72 hpf. JB-4 serial sectioning was conducted on three WT and three *ocn*−/− embryos to examine the anterior pronephros; the location is marked by the dashed vertical line. WT siblings had an intact pronephros (dotted outline), including a glomerulus (asterisk) with two tubules. Mutant sections of this same region had no discernable blood filter or tubule structure. Scale bar is 50 μm. (**C**) At 48 hpf, *ocn*::cdh17::GFP embryos were injected with 70 kDA rhodamine dextran (red). These embryos were assessed at 96 hpf. Nephron tubules are shown by the dotted outline. WT siblings exhibited no edema and appeared to uptake the dextran in the proximal region, as indicated by yellow coloration (inset). However, in mutants with pericardial edema and truncated tubules, there was no evidence of dextran within the tubule, suggesting that active uptake was not occurring in these mutants. Scale bar is 15 μm for lateral images and 50 μm for dorsal views. (**D**) After assessment of the genetic candidates obtained via whole genome sequencing, *osr1* appeared to be an attractive possibility due to a C to T SNP that was predicted to cause a premature stop codon. The predicted lesion (red shape) is located in exon 2 of *osr1.* We designed primers that flanked exon 2 (arrow heads) for Sanger sequencing. Embryos with reduced *wt1a* WISH staining exhibited a “TGA” codon within exon 2 of *osr1* that is normally a “CGA” codon in WT embryos. Scale bar is 30 μm. (**E**) To confirm that *wt1a*+ podocytes were reduced in *ocn*−/−, FISH with *wt1a* and *wt1b* was performed at 24 hpf. There were little to no double-positive cells seen in genotype-confirmed mutants, whereas both clusters of *wt1a/b*+ podocytes were evident in WT embryos. Scale bar is 10 μm. (**F**) The slit diaphragm marker *nphs1* was similarly reduced in 24 hpf *ocn* mutants. Scale bar is 50 μm. (**G**,**H**) At the 15 ss, *pax2a* marks the developing IM, the beginning of which is shown with green arrowheads. In *ocn*−/−, the anterior region of *pax2a* is decreased. When *ocn*−/− was injected with *osr1* cRNA, *pax2a* expression was restored. Interestingly, *pax2a* was significantly expanded in WT embryos injected with *osr1*. Absolute area measurements of *pax2a* were taken from somites 1–5. *p*-values: ** *p* < 0.001, * *p* < 0.05, N.S. = not significant. Scale bar is 50 μm.

Despite these differences, zebrafish do exhibit fundamental genetic and morphological similarities in kidney organogenesis to mammals. For example, the mammalian renal progenitor markers *Lim homeobox 1* (*LHX1)* and *paired box gene 2 (PAX2)* are orthologous to *LIM homeobox 1a* (*lhx1a*) and *paired box 2a* (*pax2a*), which are also renal progenitor markers in zebrafish [17,18]. Zebrafish podocytes resemble their mammalian counterparts and express markers including *Wilms tumor 1a, Wilms tumor 1b, nephrosis 1, congenital Finnish type (nephrin), nephrosis 2, idiopathic,* and *steroid-resistant (podocin)* (*wt1a/b*, *nphs1,* and *nphs2)* that closely correspond to the human homologs *WT1, NPHS1,* and *NPHS2,* respectfully [19,20,21,22]. Further, the zebrafish nephron exhibits a conserved collection of solute transporter genes that are arranged into two proximal and two distal segments similar to other vertebrates including mammals (Figure 1A) [23,24,25]. The genetic conservation combined with the simplicity of the two-nephron and single blood filter pronephros makes the embryonic zebrafish kidney an accessible and powerful genetic model for gaining insight into the many puzzles and complexities of kidney development. 

A critical regulator of kidney development in both zebrafish and mammals is the zinc-finger transcription factor *odd skipped-related 1* (*osr1*); in mice, *Osr1* is one of the earliest markers of the IM. Fate-mapping studies have shown that *Osr1*+ cells differentiate into renal progenitors and renal-associated vasculature [26]. *Osr1*−/− mice fail to express renal progenitors or develop metanephric kidneys, which contributes to embryonic lethality [27,28]. Similar to mouse studies, zebrafish *osr1* is an initial marker of the IM [26]. Further, knockdown of *osr1* causes edema, disrupts glomerular morphogenesis, and reduces proximal tubules in both zebrafish and *Xenopus* [29]. Subsequent studies have confirmed these findings [10,30,31,32,33], though the intriguing observation that *osr1* knockdown causes the kidney structure to be lost in the region abutting somites 3–5 is not fully understood. In humans, mutations in *OSR1* have been clinically linked to hypomorphic kidneys, making the continued study of this factor and its genetic regulatory network a necessity [34]. 

Here, we report the zebrafish *oceanside* (*ocn*) mutation, which was identified from a forward genetic screen for defects in kidney development based on its striking reduction in podocytes and anterior pronephros tubules [35,36], a phenotype resembling other podocyte mutants [37]. Whole-genome sequencing revealed a novel causative lesion: a premature stop codon in exon 2 of *osr1.* In addition to *ocn*−/− recapitulating previously observed alterations in mesoderm-derived tissues, reductions in nephron tubules and podocytes were rescued with ectopic *osr1* cRNA. Interestingly, *osr1* was not needed to establish the renal progenitor field but was needed to maintain the renal progenitors, as they underwent apoptosis in the absence of Osr1. We also found that developing podocytes expressed *wnt2ba* and that this expression was significantly decreased in *ocn*−/−. A loss of *wnt2ba* led to a reduction in podocytes, and ectopic *wnt2ba* partially restored these cells in *ocn*−/−. Further, we placed *wnt2ba* downstream of the antagonistic influences exerted by *osr1* and *hand2* during renal progenitor ontogeny. Together, these data illuminate novel functions of Osr1 that are essential for forwarding our understanding of kidney development and may have important implications for congenital renal defects and diseases.

## 2. Materials and Methods

### 2.1. Creation and Maintenance of Zebrafish Lines

Zebrafish were housed in the Center for Zebrafish Research in the Freimann Life Science Center at the University of Notre Dame. All experiments and protocols used in this study were approved by the Institutional Animal Care and Use Committee (IACUC) with protocol numbers 16-07-325 and 19-06-5412. We performed an ENU haploid genetic screen as described [35,36]. 

### 2.2. Live Imaging and Dextran Injections

Embryos were grown in E3 medium at approximately 28 °C. For live imaging, embryos were placed in a solution of 2% methylcellulose/E3 and 0.02% tricaine and placed in a glass depression slide. For dextran injection experiments, embryos were also incubated with 0.0003% phenylthiourea (PTU) in E3 to inhibit pigment development. At 3 dpf, *ocn*::*cdh17*::GFP animals were anesthetized and injected with 40 kDA rhodamine-dextran. Embryos were then examined and imaged 24 h after injection. 

### 2.3. WISH, FISH, IF, Sectioning and Image Acquisition

WISH was performed as described in previous studies [23,24,38,39,40]. For each marker, embryos from at least 3 sets of adult parents (e.g., 3 biological replicates) were assessed, and a minimum 5 mutants and 5 siblings were imaged for each experiment. FISH was performed as described [41,42]. Immunofluorescence (IF) was performed as previously described [37,42]. Embryos from WISH experiments were embedded in JB-4 plastic blocks and cut to obtain 4 μm sections that were counterstained with methylene blue (0.5%). Alcian blue staining was performed as described [36].

### 2.4. Genotyping

Direct genotyping on *ocn* fin clips and embryos was carried out by PCR amplification of exon 2 of *osr1*: forward primer: CCCCATTCACTTTGCCACGCTGCACCTTTTC, reverse primer: CTGTGGTCTCTCAGGTGGTCCTGCCTCCTAAA). Dilutions of purified PCR products were then subjected to Sanger sequencing by the Genomics Core at University of Notre Dame using the forward primer. 

### 2.5. Morpholinos and RT-PCR

*osr1* morpholino (ATCTCATCCTTACCTGTGGTCTCTC) was first described in [30] and was designed to block the splice donor site of exon 2. We used the primers GTGACTGTATCTGAATCCTCTTATTTTGGATCGTCTCGCTTCACAAAGAACTG and CTGTAGGCTATGGAAGTTTGCCTTTTCAGGAAGCTCTTTGGTCAG to perform RT-PCR as described in [37] to confirm the interruption of exon 2 splicing activity. *wnt2ba* splice-blocking morpholino (CTGCAGAAACAAACAGACAATTAAG) was previously utilized in [32], and the following primers were used to amplify the entire transcript by RT-PCR: forward primer: ATGCCAGAGTGTGATGGAGTTGGGTGCGCGTCGCCGGCGC, reverse primer: GCTGGAGCGAGACCACACTGTGTTCGGCCGC, and additionally looked for the presence of intronic sequence with intronic forward primer: ATCACAGGGGTATCATTATCACAAAAAATTGTAAATAAATG. While this splice-blocking morpholino was the primary method of *wnt2ba* knockdown, a *wnt2ba* ATG morpholino, ACCCAACTCCATCACACTCTGGCAT [43], was used to confirm phenotypes seen with the splicing morpholino. The *hand2* ATG morpholino (CCTCCAACTAAACTCATGGCGACAG) was used as described in [44].

### 2.6. Statistics and Measurements

Absolute domain lengths and area measurements were taken from five representative embryos per control or experimental treatment group, performed in triplicate using Fiji ImageJ. Averages, standard deviations, and unpaired Student’s *t*-tests were then calculated in Microsoft Excel and GraphPad Prism. In experiments where *osr1* cRNA was used, body axis measurements were taken for injected and uninjected embryos. The tubule measurements for each group were divided by the body length to discern what percentage of the body length was occupied by the kidney. To normalize the data, these percentages were subjected to arcsine degree transformation and then run through a Student’s *t*-test to determine significance.

## 3. Results

### 3.1. ocn Encodes a Premature Stop Codon in osr1 and Mutants Exhibits Defective Podocyte and Pronephric Tubule Development

A forward genetic haploid screen was performed to identify regulators of nephrogenesis using the zebrafish pronephros model [35,36]. The *ocn* mutant was isolated due to its loss of podocytes and abrogation of the anterior pronephros (Figure 1A). Whole-mount in situ hybridization (WISH) was performed to delineate the two pronephros tubules based on the expression of transcripts encoding *cadherin 17* (*cdh17*) and the podocytes based on *wt1b* expression at 24 and 48 h post fertilization (hpf) (Figure 1A). Both tubule length and podocyte area were significantly reduced in *ocn* mutants at these time points compared with wild-type (WT) embryos (Supplemental Figure S1A). By 72 hpf, *ocn* mutants exhibited dramatic pericardial edema that progressed in severity through 120 hpf and was ultimately lethal (Figure 1B and Figure S1B). Since the kidneys play a major role in fluid homeostasis, this phenotype was a probable indicator of renal dysfunction. 

To explore this further, WISH staining to assess tubule and podocyte morphology was conducted on 72 hpf *ocn* and WT embryos. The animals were embedded in JB-4 plastic resin and serially sectioned. In WT embryos, the blood filter could be detected as a mass of dense capillaries containing glomerular podocytes (*wt1b+*) that were flanked by *cdh17+* tubules (Figure 1B). In *ocn* mutants, however, both the midline glomerulus structure and the flanking proximal tubules were abrogated (Figure 1B). Instead, a dilated dorsal aorta was identified in this region (Figure 1B). While it was clear that the proximal pronephros was absent in *ocn* mutants, it was uncertain if this truncated kidney retained any functionality. Therefore, kidney functionality was assessed using an endocytosis assay whereby 70 kDA rhodamine-dextran was injected into the vasculature of *ocn*::*cdh17*::GFP embryos, which exhibited a pronephric truncation that phenocopied WISH experiments at 3 days post fertilization (dpf) onwards (Supplemental Figure S1C). Transgenic animals were injected with rhodamine-dextran at 48 hpf and then assessed at 48 h post injection (hpi). While dextran was endocytosed in the proximal tubules of non-edemic WT siblings, there was no dextran uptake observed within the truncated tubules of the edemic *ocn* mutant embryos (Figure 1C). Additionally, we assessed epithelial polarity through the immunofluorescence (IF) staining of Na-K-ATPase, which marks these transporters localized along the basolateral sides of kidney epithelial cells, and aPKC, which marks the apical epithelial side [45]. This experiment revealed a similar reduction in tubule and podocytes in *ocn* as seen with our WISH experiments using the markers *cdh17/wt1b* (Supplemental Figure S1D). Together, this provided strong evidence that the stunted pronephros in *ocn*−/− was indeed functionally defective. 

Next, to identify the causative lesion in *ocn*, whole-genome sequencing was conducted on pools of genomic DNA collected from 24 hpf WISH-identified putative mutants and WT siblings [46,47]. The analysis of the sequencing was performed using SNPtrack software, whereby we detected a strong candidate SNP that was centrally located on chromosome 13 (Supplemental Figure S2A). Specifically, the putative SNP encoded a missense C to T mutation and was predicted to result in an amino acid substitution from an arginine to a premature stop codon in exon 2 of *osr1* (Figure 1D and Figure S2A). 

To further assess if the predicted stop codon in exon 2 of *osr1* was linked with the *ocn* phenotype, we performed additional genotyping analysis. For this, genomic DNA was isolated from individual embryos that had been identified as *ocn* mutants or WTs, based on WISH with the podocyte marker *wt1a* at 24 hpf, and PCR was performed to amplify exon 2 of *osr1* followed by direct Sanger sequencing (Figure 1D). Out of 20 *ocn* embryos with reduced *wt1a* staining, all 20 were homozygous for the C to T mutation in exon 2 of *osr1* (Figure 1D). Protein alignment showed that zebrafish and human OSR1 protein are 264 amino acids (aa) and 266 aa in length, respectively (Supplemental Figure S2B). While they exhibit 77% conservation in overall aa sequence, the three zinc-finger domains responsible for DNA binding activity are 100% conserved across humans, mice, and zebrafish (Supplemental Figure S2B). The *osr1* genetic lesion in *ocn*−/− placed a premature stop codon at residue 165 before all three zinc-finger domains (Supplemental Figure S2B). This suggested that the truncated Osr1 protein produced in *ocn*−/− would not contain any functional domains and would thus be unable to act as a targeted transcription factor. Next, we verified the effectiveness of a splice-blocking *osr1* morpholino with RT-PCR (Supplemental Figure S3). The *osr1* morphants had a decrease in podocytes and proximal tubule length that phenocopied *ocn*−/− and was consistent with previously reported phenotypes [10,30,31,32] (Supplemental Figure S3).

Previous literature has indicated that *osr1* acts to restrict venous development in order to promote other mesodermal fates such as the kidney and the pectoral fins [10,30,32]. At 4 dpf, Alcian blue staining indicated that *ocn*−/− possessed shorter, malformed pectoral fins (Supplemental Figure S1E). The fin bud area, which gives rise to pectoral fins, was significantly reduced in *ocn*−/− mutants compared with siblings as seen by the marker *MDS1 and EVI1 complex locus* (*mecom*) at 24 hpf (Supplemental Figure S1F). Additionally, Alcian blue staining revealed altered craniofacial cartilage formation in mutants, which fits with previous literature placing *osr1* as a regulator of palatogenesis in zebrafish and mice [48,49] (Supplemental Figure S1E). In sum, these mesodermal phenotypes were consistent with *osr1* deficiency. 

Next, we evaluated other aspects of pronephros development. As *wt1a* expression appeared to be severely diminished and also disorganized in *ocn*−/−, we evaluated additional markers to better understand the features of podocyte lineage development in mutants. Podocytes were examined at 24 hpf using a *wt1a/wt1b* double fluorescent in situ (FISH). While clusters of *wt1a+/wt1b+* podocytes were visible in siblings, mutants had a scarcity of double-positive cells (Figure 1E). There was also a dearth of *nphs1+* cells, which is a marker of the podocyte slit diaphragm and suggested that podocyte differentiation was also disrupted (Figure 1F). Additionally, *ocn*−/− embryos displayed diminished *pax2a* expression at the 15 ss compared to siblings (Figure 1G), a characteristic of *osr1* morphants in previous studies as well [10,30,32]. 

To test whether the mutation in *osr1* was the specific cause of this phenotype, we performed rescue studies. Injection with *osr1* capped RNA (cRNA) rescued this domain in *ocn* mutants and expanded it in WT siblings (Figure 1G,H). In sum, *ocn* mutants reciprocated a multitude of mesodermal phenotypes seen in *osr1* literature in zebrafish and across taxa. Given the ability of *osr1* cRNA to rescue key mesodermal phenotypes in *ocn*−/− and the catastrophic nature of the *osr1* mutation, we concluded that Osr1 deficiency is responsible for the *ocn* phenotype.

### 3.2. Kidney Progenitors Are Specified in osr1 Deficient Animals, but Subsequently Undergo Apoptosis 

Previous studies suggest that the anterior pronephros abrogation in *osr1* zebrafish morphants is due to a fate change where blood/vasculature and endoderm form instead of renal progenitors [30,33,50]. Interestingly, in the *Osr1* mouse knockout model, there was an increase in apoptosis that occurred within the kidney tissue [27]. However, in both models, renal progenitors are initially established [27,30]. Thus, we next sought to delineate the cellular dynamics of renal progenitor specification in our *ocn* mutant model and to address if alterations in proliferation or apoptosis occur during pronephros development in the absence of *osr1*. 

**Figure 2:**
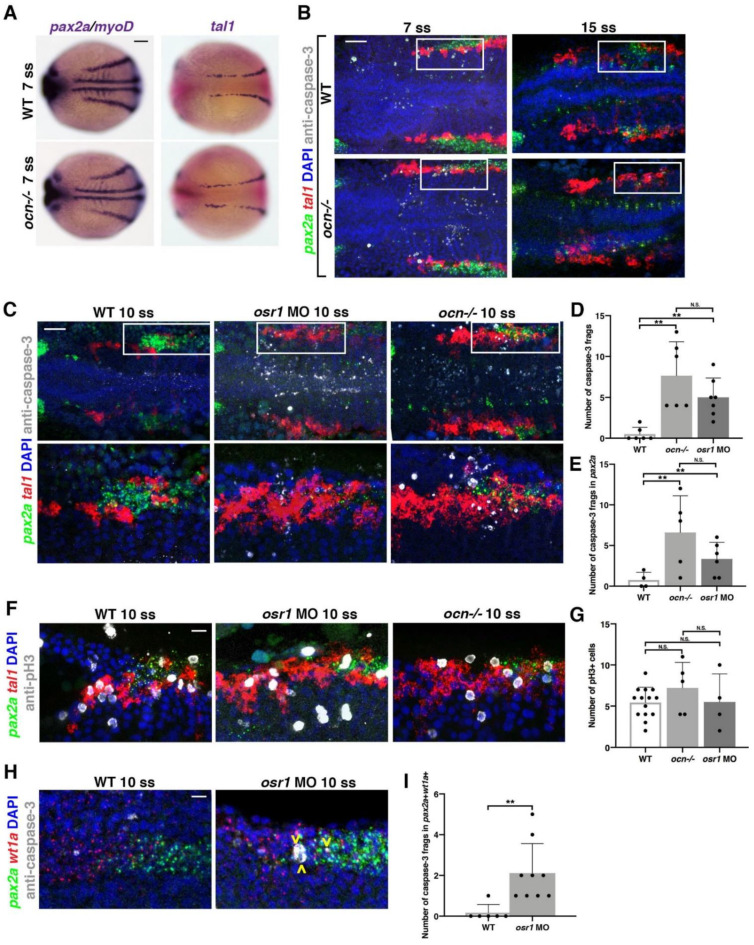
*osr1* is required to maintain and promote kidney development at the expense of hemangioblasts. (**A**) Although *pax2a* is restricted in 15 ss mutants, at 7 ss, *ocn−/−* embryos had a *pax2a* domain that appeared to occupy the same domain as WT siblings. Similarly, the hemangioblast marker *tal1* appeared mostly WT in *ocn−/−* at 7 ss. Scale bar is 50 μm (**B**) FISH with probes for *pax2a* (green) and *tal1* (red) and ICC using anti-caspase-3 (white) to mark apoptotic cells was conducted at 7 ss and 15 ss. The number of fragments in the combined *pax2a* and *tal1* fields from somites 1–5 were increased in mutants at 7 ss but not at 15 ss. Scale bar is 35 μm (**C**–**E**) At 10 ss, little to no caspase-3 fragments were seen in *pax2a* or *tal1* domains from somites 1–5, but a significant number were seen in *osr1* morphants and *ocn−/−*. The *tal1* domain was also expanded in both loss-of-function models. Scale bar is 35 μm. (**F,G**) ICC with the proliferative cell marker anti-pH3 was also conducted. Despite the expansion in *tal1* in *osr1*-deficient models, there was no significant change in the number of proliferating cells. Scale bar is 10 μm. (**H**,**I**) FISH experiments were conducted to assess changes in apoptosis in *wt1a+/pax2a+* podocyte progenitors. There was a significant increase in the number of apoptotic fragments within this field in mutants compared with WT siblings. Scale bar is 10 μm. A minimum of three individuals were assessed for each group across experiments. Photos are max intensity projections from z-stacks, and each side of mesoderm was quantified individually. *p*-values: ** *p* < 0.001, N.S. = not significant.

To investigate this, we first performed WISH studies. The LPM is marked by *T-cell acute lymphocytic leukemia 1* (*tal1*), and gives rise to hemangioblasts [51,52]. The IM and hemangioblast domains at the 7 ss were not noticeably different between WT and *ocn−/−* embryos, as indicated by the markers *pax2a*, and *tal1* (Figure 2A). However, as previously noted, by the 15 ss, there was a decrease in the anterior-most domain of *pax2a* expression in *ocn−/−* embryos (Figure 1G). To further assess the anterior *pax2a*+ cells between the 7 ss and 15 ss, we performed double FISH studies in WT and *ocn−/−* embryos to assess *pax2a* and *tal1* expression. DAPI staining was also utilized to discern features such as the trunk somites, which allowed for accurate staging. Embryos were flat-mounted and imaged as previously described (Supplemental Figure S2A) [39]. Further, IF was also performed on these samples with anti-caspase-3 antibody to assess the number apoptotic bodies or anti-pH3 to identify proliferating cells. In our analysis, we focused on the changes to these markers within somites 1–5, as the IM adjacent to somite 3 gives rise to podocytes [21].

Beginning at the 7 ss, *ocn−*/*−* embryos exhibited a significant increase in the number of caspase-3+ fragments within the combined *tal1* and *pax2a* fields near somites 1–5 (Figure 2B). However, by the 15 ss, few apoptotic fragments were visible in the area of interest, with no significant differences between WT and *ocn−/−* embryos (Figure 2B). Similar to the 7 ss, we found a significant increase in the number of total caspase-3+ fragments at the 10 ss in *ocn−/−* mutants and *osr1* morphants, while the WT siblings had little to no apoptosis occurring in this area (Figure 2C–E). Interestingly, most of the apoptosis that occurred in mutants and morphants happened within the *pax2a* kidney field specifically (Figure 2E). Another finding of note was that the caspase-3+ fragment number was not significantly different between *osr1* morphants and *ocn−/−* for either assessment (Figure 2D,E). 

To further understand the cell dynamics across this time course, absolute area measurements of *pax2a* and *tal1* were taken at 7, 10, and 15 ss from somites 1–5 in WT and *ocn−/−*. Surprisingly, the area of the *tal1* domain was already expanded at 7 ss and continued to expand through the 15 ss (Supplemental Figure S4). However, across the 3 time points examined, a reduction in *pax2a* area was only significantly different between WT and *ocn−/−* at 15 ss (Supplemental Figure S4). Further, although the *tal1* field was expanded in *ocn−/−* embryos at the 10 ss, there was no significant difference in proliferating pH3+ cells between WT and *osr1* loss of function models (Figure 2F,G). Additionally, no significant changes in proliferation were seen between mutants and WTs at the 8 ss (Supplemental Figure S4).

To determine if apoptosis was occurring within podocyte progenitors in the *pax2a* kidney field, we performed an additional FISH with *wt1a* and *pax2a* at 10 ss. During this time point, while *pax2a* expression begans adjacent to somite 3, *wt1a* was expressed from somites 1–3 (Figure 2H). Similar to the *pax2a* domain, the *wt1a* domain did not appear to be reduced at this time point, though it did become restricted and disorganized by 24 hpf (Figure 1D). We found a significant increase in caspase-3+ fragments that were double-positive for *wt1a* and *pax2a* in *osr1* morphants compared with WTs (Figure 2H,I). These results demonstrated that abnormal apoptosis occurred in podocyte progenitors due to the loss of *osr1*. In sum, *osr1* is not needed to initiate the *pax2a* progenitor pool, but it is needed to maintain this population, including the podocyte precursors, during pronephros development. 

### 3.3. Ectopic osr1 Is Transiently Sufficient to Rescue Renal Progenitors 

Our observation that *pax2a+* renal progenitors arise in *ocn* mutants, but are not maintained, is consistent with previous data that *osr1* knockdown leads to a reduced *pax2a+* renal progenitor field by the 14 ss [30]. As *pax2a* expression marks both podocyte and tubule precursors [31], we hypothesized that *osr1* is likely needed for podocyte and tubule progenitor maintenance. To determine this, we performed a series of rescue studies in our *ocn* mutants to test if one or both of these compartments requires *osr1* for its maintenance. 

**Figure 3:**
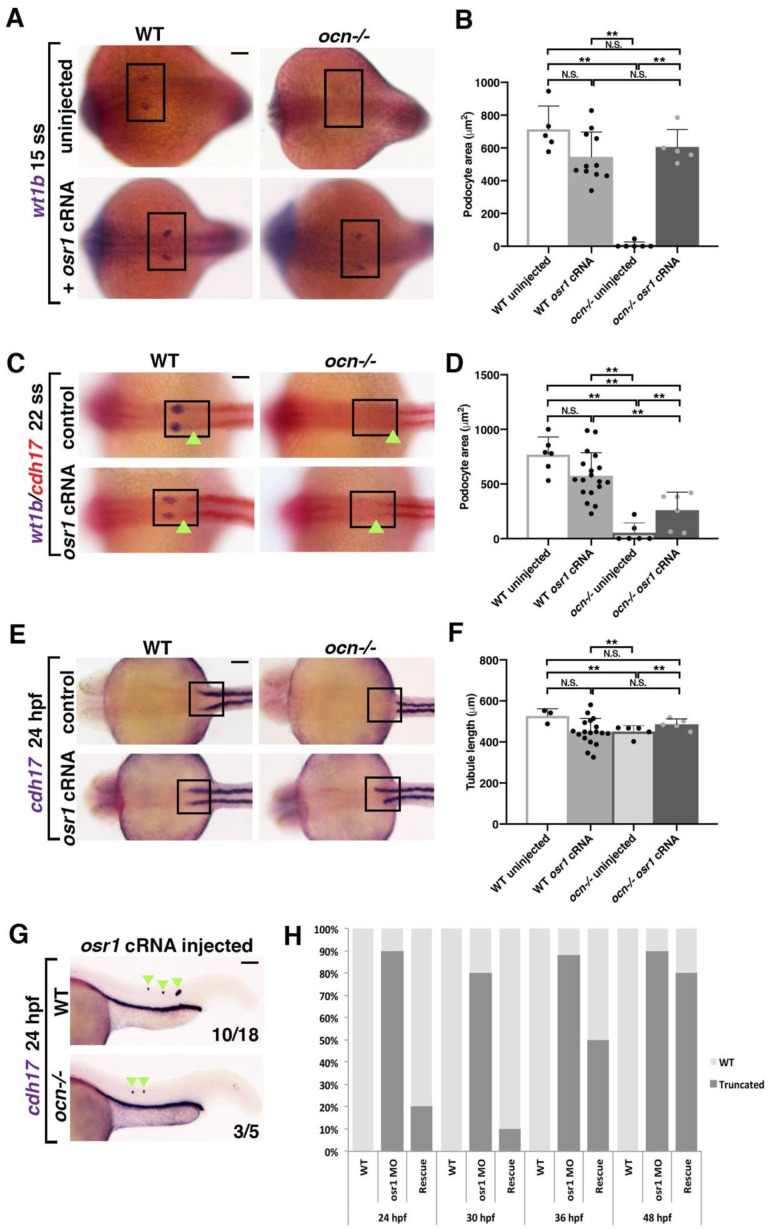
*osr1* is required for the continued development of kidney lineages. (**A**,**B**) Embryos from *ocn* incrosses were injected at the 1-cell stage with 50 pgs of *osr1* cRNA and examined. At 15 ss, podocytes (*wt1b*) were robustly rescued in *ocn−/−*. (**C,D**) However, by 22 ss, podocytes were only partially rescued in *ocn−/−* injected embryos, while tubule lengths in the same animals were not significantly different from WTs, as shown by the green arrowheads. (**E**–**G**) Truncated tubule was still able to be rescued in mutants at 24 hpf. Further, overexpression of *osr1* induced ectopic tubule formation (green arrowheads). (**H**) A rescue time course was conducted with *osr1* MO and *osr1* cRNA to determine when the *osr1* cRNA dosage became insufficient to rescue. While there was a 90% rescue rate at 24 hpf, by 48 hpf, this rate had dropped to 20%. This indicated that continued *osr1* is needed for normal tubule development. *p*-values: ** *p* < 0.001, N.S. = not significant. For tubule rescue at 24 hpf, p-values were obtained from arcsin transformed kidney to body percentage calculations for each group. Scale bar is 50 μm for all images.

First, we tested whether the overexpression of *osr1* mRNA was sufficient to rescue podocytes in *ocn* mutants by assessing *wt1b* expression, which specifically marks the podocyte lineage [20,21]. The provision of *osr1* mRNA robustly rescued the development of *wt1b+* podocytes in *ocn* mutants at the 15 ss (Figure 3A,B). However, by the 22 ss, we were only able to achieve a partial podocyte rescue, though tubules within the same individuals appeared to be WT in length (Figure 3C,D). Consistent with this, we were unable to obtain a podocyte rescue at 24 hpf (data not shown), though again we could achieve a rescue of the truncated tubules (Figure 3E,F). Interestingly, the overexpression of *osr1* was sufficient to induce ectopic *cdh17+* cells in about 55% of injected embryos (Figure 3G). It should be noted that *osr1* cRNA did lead to a decrease in body axis length when compared with uninjected WTs and mutants, which in turn affected pronephros length (Supplemental Figure S5). Despite this, the percentage of kidney length to body length was not significantly different between embryos injected with *osr1* cRNA and uninjected animals. 

We also conducted a rescue time course by co-injecting *osr1* MO and *osr1* cRNA and performed WISH using *cdh17* to assess the tubules during a number of stages. While 90% of animals injected with both constructs exhibited a WT tubule length at 24–27 ss, by 36 hpf, only 50% showed a rescue (Figure 3H and Figure S5B). At 48 hpf, only 20% of injected embryos had a WT length pronephric tubules while 80% had a unilateral or bilateral reduction (Figure 3H and Figure S5B). Together, this indicated that the pronephros requires a continued presence of *osr1* to maintain the tubule population as development progressed.

### 3.4. wnt2ba Is a Novel Podocyte Marker and Regulator 

**Figure 4:**
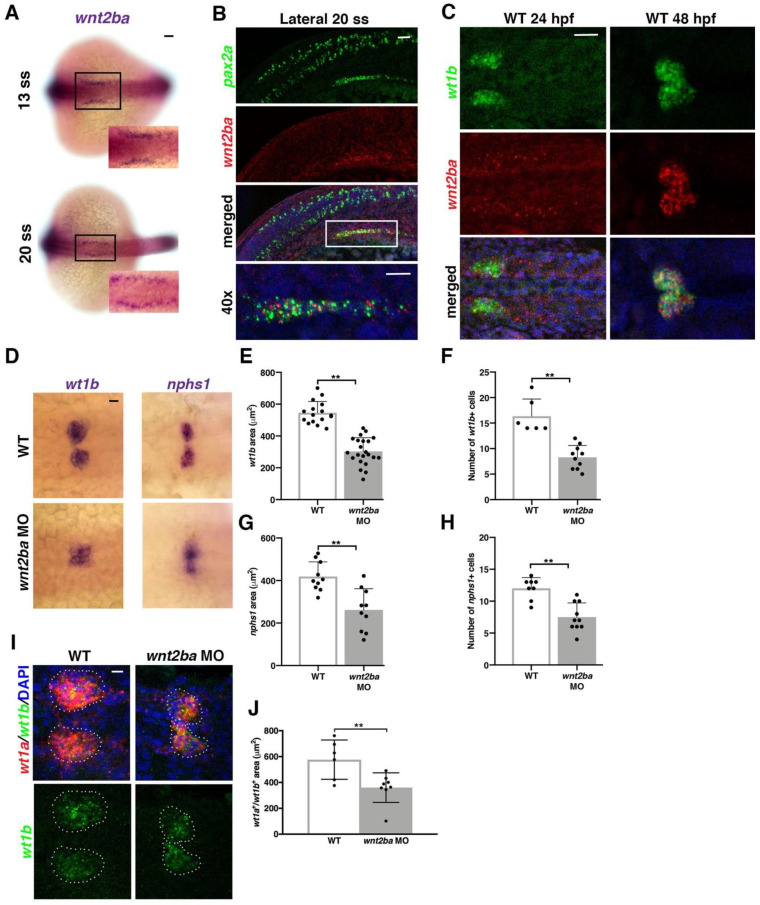
*wnt2ba* is a podocyte marker and regulator. (**A**) *wnt2ba* is expressed in bilateral stripes as early as 13 ss. Scale bar is 30 μm. (**B**) *wnt2ba* (red) is expressed within the anteriormost region of the IM, as shown by colocalization with *pax2a* (green). White box denotes area of colocalization, which is magnified in bottom panel. DAPI (blue) marks nuclei. Scale bar is 15 μm. (**C**) *wnt2ba* (red) also colocalized with the podocyte marker *wt1b* at 24 hpf, though at this time point it was also expressed in the putative neck segment domain. By 48 hpf, the *wnt2ba* domain was specified to the podocytes. Scale bar is 30 μm. (**D**–**H**) Podocyte area and cell number was assessed in *wnt2ba* morpholino-injected animals and determined to be reduced compared with WT controls. Both *wt1b* and *nphs1* showed a significant decrease in domain area in *wnt2ba* morphants compared with WT embryos. (**I**,**J**) FISH with *wt1a* and *wt1b* was performed at 24 hpf. There was a significant area reduction in the podocyte domain seen in *wnt2ba* morphants compared to WTs. Scale bar is 10 μm. *p*-values: ** *p* < 0.001. Scale bar is 30 μm.

Given the importance of *osr1* to podocyte development and maintenance, we wanted to identify downstream factors that promote podocytes. It was previously shown that the canonical Wnt ligand *wingless-type MMTV integration site family, member 2Ba* (*wnt2ba*) is expressed in a similar proximal swath of IM as *osr1* [32]. We observed a similar expression pattern of *wnt2ba* in the anterior IM as early as 13 ss (Figure 4A). To specifically determine which cells *wnt2ba* was expressed in, we conducted FISH studies. At the 20–22 ss, *wnt2ba* transcripts were colocalized in cells within the anterior most region of *pax2a+* and *wt1b+* IM (Figure 4B and Figure S6). At 15 ss, *wnt2ba* transcripts were also colocalized with *wt1a/b+* podocyte progenitor cells (Supplemental Figure S6). At 24 hpf, *wnt2ba* was expressed in both *wt1b*+ podocyte precursor cells and in the neighboring cells of the IM (Figure 4C). By 48 hpf, *wnt2ba* was restricted to the podocytes and overlapped precisely with *wt1b* expression (Figure 4C). Taken together, we conclude that *wnt2ba* is a novel podocyte marker, thereby extending prior observations [32]. We also examined the expression of the zebrafish *wnt2ba* paralogue *wnt2bb* at 24 hpf using FISH and determined that these transcripts by comparison were located anterior to both the podocyte and kidney fields (Supplemental Figure S7).

Given its expression in podocyte progenitors, we hypothesized that Wnt2ba might have roles in podocyte specification or differentiation. To explore whether *wnt2ba* is needed for proper podocyte formation, we performed *wnt2ba* loss of function studies. We first verified a morpholino that blocked splicing at exon 1, as well as a morpholino that targeted the start site (Supplemental Figure S8). When *wnt2ba* morphants were examined at 24 hpf, there was a significant reduction in the expression of *wt1b* and *nphs1* that corresponded to a smaller podocyte area and net cell number (Figure 4D–H). We also found that the area of *wt1a+/wt1b+* co-expressing podocytes was decreased in *wnt2ba* morphants at 24 hpf (Figure 4I,J). Furthermore, the decrease in podocyte number in *wnt2ba* morphants occurred between the 15 and 22 ss, suggesting *wnt2ba* is required for maintaining the podocyte lineage (Supplemental Figure S9). In contrast, *wnt2ba* morphants showed no discernable changes in the development or maintenance of the *cdh17*+ nephron tubule (Supplemental Figure S8E). Collectively, these data lead us to conclude that *wnt2ba* is a significant regulator of podocyte ontogeny. 

### 3.5. osr1 Promotes wnt2ba in the Podocyte Developmental Pathway

Previous research has demonstrated that *osr1* morphants exhibited a dramatic decrease in the *wnt2ba* pronephric domain, though *wnt2ba* morphants had no notable change in *osr1* expression [32]. They postulated that *osr1* acts to promote *wnt2ba* in the IM, so which allows for proper pectoral fin development to occur [32]. This led us to hypothesize that this same genetic cascade in the IM promotes the formation of proximal pronephric tissues, such as the podocytes, and is dysfunctional in *ocn−/−.*

**Figure 5:**
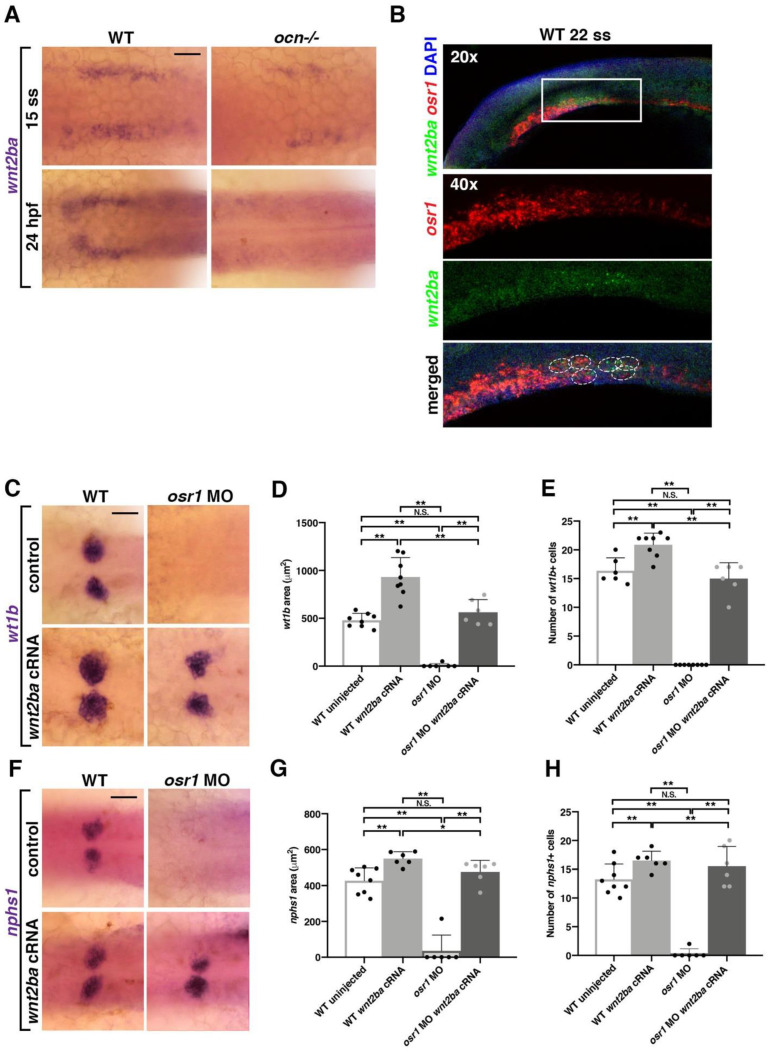
*wnt2ba* is sufficient for podocyte development downstream of *osr1*. (**A**) When *wnt2ba* was assessed at 15 ss, in *ocn−/−* and WT siblings, it was evident that staining was reduced in mutants. By 24 hpf, *wnt2ba* staining was almost completely absent, tracking with the loss of other podocyte markers in *ocn−/−*. Scale bar is 30 μm. (**B**) FISH experiments showed that *osr1* and *wnt2ba* colocalized in cells at 22 ss. (**C**–**H**) Embryos were injected with *osr1* MO and/or *wnt2ba* cRNA at the one-cell stage, and podocytes and the developing slit diaphragm were visualized at 24 hpf using *wt1b* and *nphs1*, respectively. Embryos injected with *osr1* MO alone showed few podocyte or slit diaphragm cells, while embryos injected with *wnt2ba* cRNA alone had an increased podocyte area. Coinjected embryos had a partial rescue of podocytes, indicating that *wnt2ba* is a downstream factor in the podocyte pathway. A minimum of 5 individuals were imaged for quantification. *p*-values: ** *p* < 0.001, * *p* < 0.05, N.S. = not significant. Scale bar is 30 μm.

In congruence with this prior study, we found that *wnt2ba* is significantly reduced in *ocn−/−* at both 15 ss and is almost completely absent by 24 hpf (Figure 5A). We also observed that *wnt2ba* and *osr1* transcripts were colocalized in a population of presumptive IM cells at 15 ss and 22 ss, putting them in the right place and the right time to interact (Figure 5B and Figure S10). The overexpression of *wnt2ba* led to an increase in podocyte number and domain area in injected WT embryos, as seen with an increase in the markers *wt1b* and *nphs1* (Figure 5C–H). While injection with *osr1* MO alone leads to diminished podocytes, coinjection of *wnt2ba* cRNA with *osr1* MO led to a rescue in podocyte area and cell count (Figure 5C–H). Together, this indicates that *wnt2ba* is sufficient to drive podocyte development and does so downstream of *osr1.*

### 3.6. hand2 Suppresses Podocyte Development by Restricting wnt2ba Expression and Podocyte Development

The bHLH transcription factor *heart and neural crest derivatives expressed 2* (*hand2*) has been shown to be antagonistic to *osr1* in early mesoderm development [10]. The loss of *osr1* leads to decreases in podocytes and tubules and an increase in hemangioblasts; in contrast, the loss of *hand2* results in expansions in renal cells at the expense of vasculature [10]. The concomitant knockdown of *osr1* and *hand2* rescues tubule development [10] and podocyte development [33]. 

**Figure 6:**
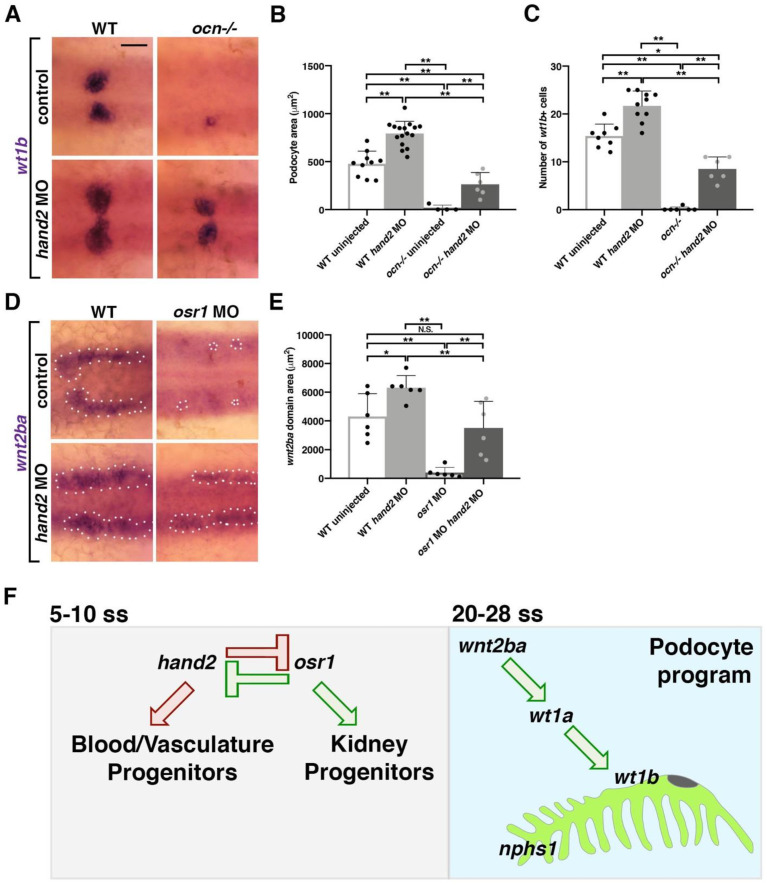
Acting in opposition to *osr1, hand2* inhibits *wnt2ba*-driven podocyte development (**A**–**C**) Embryos from *ocn* incrosses were injected with *hand2* MO. Podocyte area and cell number were partially rescued in *ocn−/−* injected embryos and were expanded in WT injected siblings. This signified that *hand2* suppresses podocyte formation. (**D**,**E**) Similarly, *wnt2ba* expression was rescued in *osr1/hand2* morphants compared with uninjected WT controls. Injection of *hand2* MO alone led to a significant increase in the *wnt2ba* domain. All images were at 24 hpf, scale bar is 30 μm. A minimum of 5 individuals were imaged for quantification. *p*-values: ** *p* < 0.001, * *p* < 0.05, N.S. = not significant. (**F**) *osr1* promotes podocyte and kidney lineages and suppress blood and vasculature, while *hand2* acts in opposition. Imbalance of either of these factors leads to changes in mesodermal fates. Podocyte development is one example of a mesodermal fate that is altered by imbalance of *osr1/hand2.* This is because the downstream factor *wnt2ba* is decreased without *osr1* yet increased in the absence of *hand2*. *wnt2ba* endorses the podocyte factor *wt1a/b,* which has been shown to be required for formation of podocytes and the slit diaphragm (*nphs1*).

When we knocked down *hand2* using an ATG morpholino, we observed a separation in the *myosin light chain 7* (*myl7*) heart field at 22 ss that matched previously observed phenotypes [44] (Supplemental Figure S11). The knockdown of *hand2* also caused a significant increase in *wt1b*+ podocyte domain area and cell number (Figure 6A–C). While uninjected *ocn−/−* embryos had few to no podocytes, injecting *ocn-/-* with *hand2* MO resulted in an expansion in podocyte number and area that was significantly different from mutants (Figure 6A–C). Similarly, *hand2* morphants had a significantly larger *wnt2ba* domain, and *hand2/osr1* MO coinjection rescued the usually abrogated *wnt2ba* domain (Figure 6D,E). This indicated that an imbalance of *hand2* and *osr1* leads to changes in *wnt2ba* expression, which alters podocyte development.

## 4. Discussion

While dozens to hundreds of podocyte diseases and maladies have been characterized, the genetic explanations for their origins and progression are lacking. One reason for this is that there are relatively few factors that are known to promote the development of these specialized epithelia. Continuing to identify these factors is critical for future diagnostics and treatments for podocytopathy. In this study, we have both reexamined a previous factor shown to promote podocyte fates, *osr1*, and also identified a new downstream regulator, *wnt2ba*. 

In this study, *ocn* was identified as a mutant of interest in a forward genetic screen due to displaying pericardial edema and decreases in podocytes and proximal tubules. Through whole-genome sequencing, we determined that *ocn−/−* harbors a SNP in exon 2 that leads to a premature stop in *osr1*. This SNP was confirmed as the causative lesion in *ocn−/−* when *osr1* cRNA could rescue each of these phenotypes. Upon confirmation that *ocn* was an *osr1* mutant line, we next sought to fully assess how *osr1* loss of function impacts kidney development in the context of a zebrafish mutant. We found that *osr1* is needed to maintain renal progenitors and inhibit the development of hemangioblasts. 

Further, we established a genetic pathway controlled by *osr1* that regulates podocyte survival by promoting *wnt2ba* expression. We found that *wnt2ba* is an IM/podocyte marker that is likewise diminished in *ocn−/−*. Loss and gain of *wnt2ba* lead to a decrease and increase in podocyte area, demonstrating that *wnt2ba* is both necessary and sufficient to drive podocyte development. Notably, *wnt2ba* can rescue podocytes in an *osr1*-deficient background, which places this factor downstream of *osr1* (Figure 6F). Finally, the *osr1/wnt2ba* podocyte pathway is negatively regulated by *hand2* (Figure 6F). 

### 4.1. Osr1 Acts to Promote Podocytes

The earliest known podocyte marker in zebrafish is *wt1a,* though the paralogue, *wt1b,* that appears at 12 ss is expressed in a more specific territory [20,53]. It has also been suggested that *wt1a* is more dominant than *wt1b*, as knockdown of *wt1a* leads to loss of *nphs1/2*, while knockdown of *wt1b* causes less dramatic podocyte phenotypes [54]. Zebrafish literature has shown that *osr1* morphants exhibit reductions in *wt1b*, *lhx1a,* and *nphs1/2* at 24 hpf that have also been observed in *ocn−/−* [29,30,31]. However, the relationship between *wt1a* and *osr1* has yet to be fully understood. Tomar et al. [31] placed *wt1a* upstream of *osr1* due to *osr1* being reduced in *wt1* morpholino-injected embryos and *wt1a* expression being interpreted as unchanged in *osr1* morpholino-injected animals. However, in our studies, *osr1* morphants did exhibit alterations in *wt1a*+ cell organization and a restriction in domain that phenocopies *ocn−/−*. Mouse studies have shown that *WT1+/−; OSR1+/−* mice exhibit smaller kidneys, suggesting that these factors act cooperatively in kidney and podocyte development [55]. If *osr1* and *wt1a* did have a similar synergistic relationship in zebrafish kidney development, this would also explain reports that *wt1* morphants exhibit a loss of podocytes and proximal tubules reminiscent of *osr1* loss of function models [31]. While there are currently limitations in using anti-Osr1 antibodies in any in vivo model, progress in this area is needed in order to ascertain if *wt1a* and *osr1* are directly interacting during kidney development. 

### 4.2. Osr1 Is Needed for Kidney Cell Maintenance 

While *ocn−/−* exhibit normal patterning of IM early in development, by the time specification to pronephros is beginning to occur around 15 ss, the anterior domain is absent. Our experiments demonstrated that this is due to two events: (1) an expansion of hemangioblasts and (2) the apoptosis of podocyte progenitors in this region. Work in chick and mouse has shown that while mesonephric tissues and markers are present, apoptosis occurs within the nephrogenic mesenchyme that keeps metanephric tissues from forming in *Osr1* knockout animals [27,28]. Further, previous studies have shown that *Osr1* acts synergistically with factors such as *Wt1* and *Six2* to renew renal stem cell pools to inhibit premature differentiation and thus cell death [55]. A similar apoptosis event has not been recorded in *osr1* loss of function zebrafish models prior to this study, and we hypothesize that *osr1* plays a similar role in progenitor self-renewal in zebrafish.

The expansion in the hemangioblast domain in *osr1* morphants has been documented by other groups, where it was suggested that *pax2a*+ cells were converting to *tal*+ cells [30]. Additionally, the expansion in vessel progenitors has been reported in an *osr1* TALEN mutant [33]. However, our results add one further element to these early events, as we have captured cell apoptosis in *pax2a+* cells of *osr1* mutant embryos. Further, our studies have revealed that the timing of the *pax2a* domain decrease and hemangioblast domain increase is not equivalent. The hemagioblasts expand hours prior to the loss of the anterior IM domain. We postulate that *osr1* may inhibit hemangioblast formation either indirectly or in an independent mechanism than it uses to promote IM and podocytes. 

### 4.3. Wnt2ba Is a Novel Regulator of Podocyte Development

Finally, *wnt2ba* is a ligand that functions in the canonical Wnt/beta-catenin pathway. As a member of this pathway, *wnt2ba* acts to promote cell growth, differentiation, and migration during development. In regard to kidney development, *Wnt2b* can be detected in the kidney stroma in mice as early as E11.5 [56,57] and in humans WNT2B is expressed in fetal kidney stroma [58]. In addition, cells expressing Wnt2b promote ureteric branching in culture [56]. *Wnt2/2b* is also paramount to normal lung and pectoral fin development in both aquatic and mammalian species [32,59]. Interestingly, *osr1* has been shown to act downstream of retinoic acid signaling yet upstream of *wnt2b* in both pectoral fin development in zebrafish [32] and in lung progenitor specification in foregut endoderm in *Xenopus* [60]. However, our study has both evaluated the role of *wnt2ba* as a regulator of kidney development and placed its function downstream of *osr1* to specifically promote the podocyte lineage. Further, we show that *osr1* promotes *wnt2ba* expression during podocyte development through a mechanism involving the inhibition of *hand2*. In synchrony with our data, a recent report similarly concluded that the reciprocal antagonism between *osr1* and *hand2* is essential for the normal emergence of *wt1b+* podocyte precursors [33]. Future work to assess whether Osr1 directly binds the *wnt2ba* promotor, and the identity of other regulatory factor(s), will be absolutely critical in order to decipher the underlying molecular mechanisms of the genetic relationships reported in the present work. Furthermore, the identification of other candidate Osr1 targets will be crucial in expanding our knowledge about the roles of this critical gene.

We show in the present study that *wnt2ba* is a regulator of podocyte development but that loss of *wnt2ba* does not cause compelling changes in either the PCT segment or the nephron tubule length. Another study by the team of Lyons et al. [61] showed that the broad inhibition of Wnt signaling through the heat-shock activation of *dkk1* led to an abrogation in the zebrafish pronephros that resembles *osr1* loss of function models. Wnt ligands are highly regionalized to allow for precise regulation during tissue development [57,62]. Our findings that *wnt2ba* is restricted to the podocytes by 48 hpf could reflect regional specificity. This suggests that there are other Wnt ligands and receptors that act to regulate certain kidney lineages in zebrafish development. The loss of one or more of these factors in combination with *wnt2ba* could lead to an anterior truncation of the pronephros that resembles the experiments from Lyons et al. [61]. Future studies are needed to discern these factors and additional downstream targets of both *wnt2ba* and *osr1*.

Taken together, these results have allowed us to garner new insights into podocyte development in zebrafish. By selecting *ocn* as a mutant of interest from our ENU screen, we have discovered an *osr1* mutant and confirmed its significance in zebrafish pronephros development in an unbiased manner. We have expanded on previous findings by demonstrating that *osr1* is required to inhibit apoptosis in specified kidney precursors, and later for nephron cell maintenance. We have also ascertained new roles for *osr1* in promoting *wnt2ba* expression, which it does in part through the antagonism of *hand2*. Finally, our results show that *wnt2ba* mitigates podocyte development downstream of the *osr1/hand2* interaction. Given how little is known about CAKUT and kidney agenesis, findings from genetics studies such as the present work are crucial to furthering our understanding about the causes and solutions to these disease states. 

## 5. Conclusions

The *ocn* zebrafish mutant provides a new vertebrate genetic model to expand our understanding about the roles of *osr1* during kidney development. During the genesis of the zebrafish pronephros, a deficiency of *osr1* causes an abrogation of podocyte and proximal tubule lineages, which are specified but subsequently undergo apoptosis in the absence of *osr1*. Furthermore, we conclude that *osr1* regulates podocyte survival by promoting the expression of *wnt2ba*, a factor that is both necessary and sufficient for podocyte ontogeny. Finally, the function of *wnt2ba* in the podocyte developmental program is impacted by the antagonistic interactions between *osr1* and the transcription factor *hand2*.

## Data Availability

All data are contained in this article and the Supplementary Materials.

## Supplementary Materials

The following supporting information can be downloaded at: https://www.mdpi.com/article/10.3390/biomedicines10112868/s1, Figure S1: Additional *ocn* phenotypes; Figure S2: A genetic lesion in the *osr1* transcription factor results in truncated osr1 protein in the *ocn* mutant line; Figure S3: Verification of *osr1* splice-blocking morpholino phenotypes; Figure S4: Additional analysis of cell dynamics in *osr1*-deficient models; Figure S5: *osr1* cRNA injection additional analysis; Figure S6: *wnt2ba* colocalizes with early, developing podocytes; Figure S7: *wnt2bb* does not colocalize with podocytes; Figure S8: Assessment of *wnt2ba* MO through RT-PCR analysis; Figure S9: *wnt2ba* knockdown causes decreased podocytes at 22 ss; Figure S10: Additional stages of *wnt2ba* and *osr1* co-localization; Figure S11: *hand2* MO replicates previous studies.

## Abbreviations

*cadherin 17* (*cdh17*); *centrin 4* (*cetn4*); chronic kidney disease (CKD); *chloride channel K* (*clcnk*); congenital anomalies of the kidney and urinary tract (CAKUT); corpuscle of Stannius (CS); days post fertilization (dpf); distal early (DE) segment; distal late (DL) segment; fluorescent in situ hybridization (FISH); hours post fertilization (hpf); intermediate mesoderm (IM); interrenal gland (IR); *LIM homeobox 1a* (*lhx1a*); multiciliated cells (MCCs); N-ethyl-N-nitrosourea (ENU); *nephrosis 1, congenital Finnish type (nephrin); nephrosis 2* (*nphs1*)*, idiopathic, steroid-resistant (podocin)*(*nphs2*), *odd-skipped related transcription factor 1* (*osr1*); *odd-skipped related transcription factor 2* (*osr2*); *outer dense fiber of sperm tails 3B* (*odf3b*); *paired box 2a* (*pax2a*); proximal convoluted tubule (PCT); proximal straight segment (PST); *renal outer medullary potassium, channel 2* (*romk2*); *solute carrier family 4 (sodium bicarbonate cotransporter), member 4a* (*slc4a4a*); *solute carrier family 9, subfamily A* (*slc9a3*); *solute carrier family 12* (*slc12a1*); *solute carrier family 12 sodium/chloride transporter, member 3* (*slc12a3*); *solute carrier family 20, member 1a* (*slc20a1a*); *transient receptor potential cation channel, subfamily M, member 7* (*trpm7*); somite stage (ss); whole mount in situ hybridization (WISH); *Wilms tumor 1a* (*wt1a*); *Wilms tumor 1b* (*wt1b*); *wingless-type MMTV integration site family, member 2Ba* (*wnt2ba*); *wingless-type MMTV integration site family, member 2Bb* (*wnt2bb*); Zebrafish International Research Center (ZIRC).
