# Supplementary material for: osr1 Maintains Renal Progenitors and Regulates Podocyte Development by Promoting wnt2ba via the Antagonism of hand2
Source: Biomedicines. 2022 Nov 9;10(11):2868. doi: 10.3390/biomedicines10112868 (PMC9687957; doi:10.3390/biomedicines10112868)
Supplement: Supplementary file 1 [file biomedicines-10-02868-s001.zip › biomedicines-1977723-supplementary.pdf]

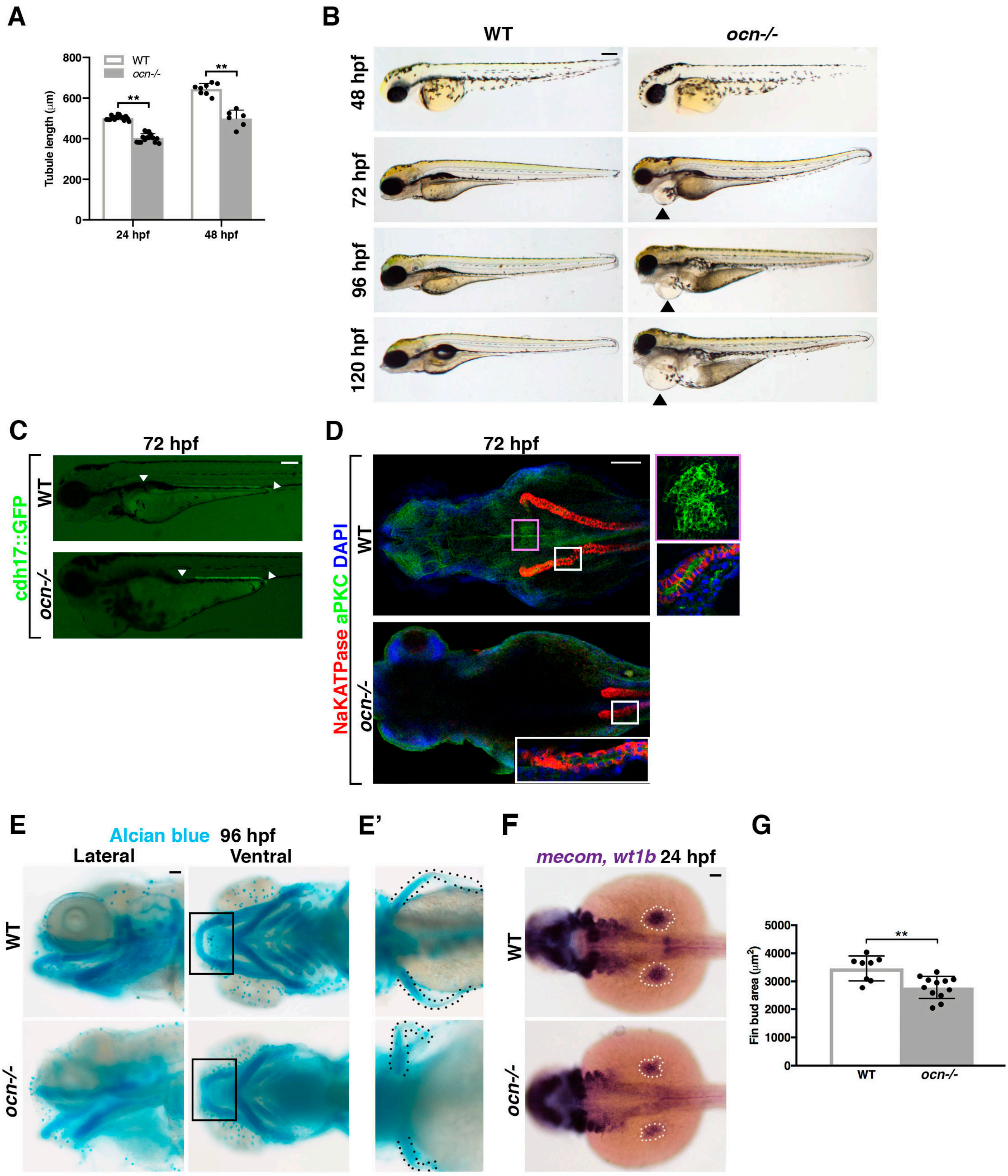

Supplemental Figure S1. Additional *ocn* phenotypes. (**A**) Conducting absolute length measurements of *cdh17* tubule revealed that *ocn* have significantly shorter nephrons tubules at 24 hpf and 48 hpf compared to WT siblings. (**B**) A live time course of *ocn-/-* revealed pericardial edema beginning at 72 hpf, as indicated by black arrow heads. Scale bar is 70 μm. (**C**) *ocn+/-* were crossed with a cdh17::GFP transgenic line to create *ocn* carriers with GFP-labeled pronephric tubules. Pictured here are 72 hpf cdh17::GFP embryos and F2 *ocn-/-*::cdh17::GFP embryos. White arrowheads demarcate the start and terminus of the pronephros, and it is evident that mutants have a shorter pronephros. Scale bar is 30 μm. (**D**) Similarly to WISH results with *cdh17* and *wt1b*, IF revealed that *ocn-/-* embryos had a truncation in NaKATPase+ tubule and absence of aPKC+ glomerulus. (**E**) The acidic stain, alcian blue, was used to show cartilage in developing *ocn-/-* and WT siblings at 96 hpf. Lateral and ventral views showed that the jaw developed in an improper orientation with unfused Meckel's cartilage (box). Scale bar is 30 μm. (**E'**)Pectoral fins in *ocn-/-* mutants were also malformed. (**F,G**) The pectoral fins arise from the fin buds (white dotted area), which are marked by *mecom* at 24 hpf. Fin bud area measurements were decreased in mutants, which could be genotyped by the absence of *wt1b+* podocytes. Scale bar is 30 μm. P-values: $^{**}p<0.001$.

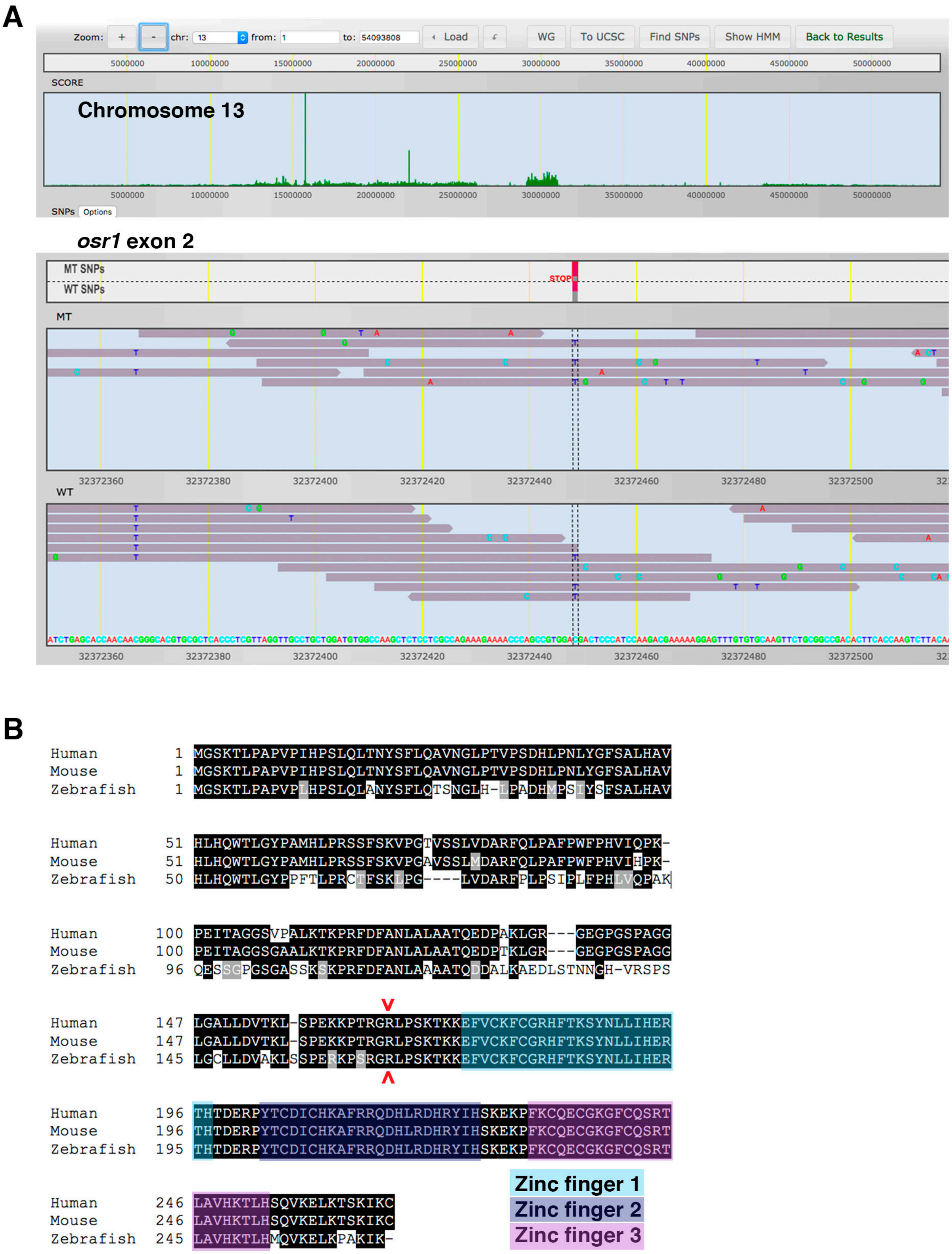

Supplemental Figure S2. A genetic lesion in the *osr1* transcription factor would result in truncated osr1 protein in the *ocn* mutant line (**A**)Whole genome sequencing was conducted on 24 hpf *ocn* mutants and WT siblings. SNPtrack analysis indicated that the genetic lesion responsible for the *ocn-/-* phenotypes was most likely located on chromosome 13. Specifically, a C to T missense mutation in exon 2 of *osr1* would cause a premature stop codon. (**B**) Zebrafish, mouse and human OSR1 protein contains three zinc-finger binding domains. The predicted SNP would result in a substitution from an arginine to a stop codon before the transcription of the zinc-finger binding domains, which are each 100% conserved across zebrafish, mice and human OSR1 protein.

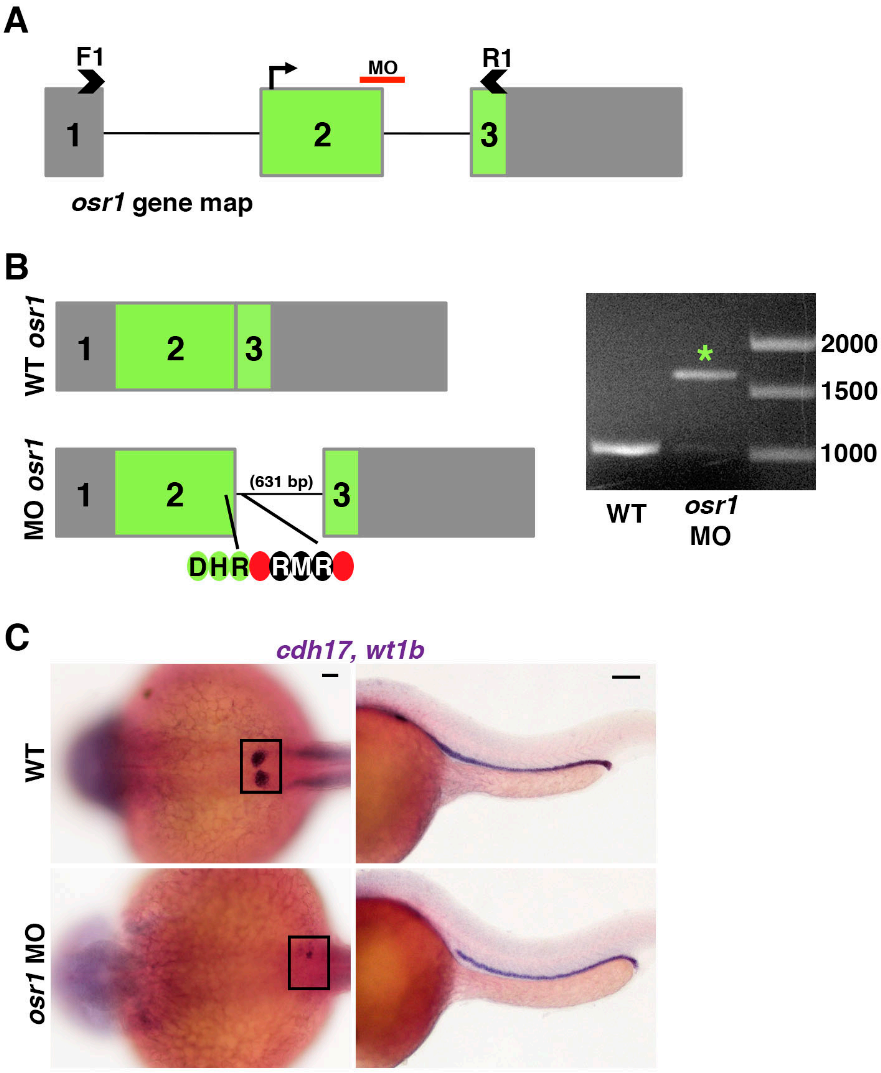

Supplemental Figure S3. Verification of *osr1* splice-blocking morpholino phenotypes. (**A,B**) A previously published morpholino was obtained to block splicing activity in exon 2 of *osr1*. Primers flanking exon 2 (arrowheads) were used to conduct RT-PCR to assess splicing activity in morphants at 24 hpf. In WTs with correct splicing, a 1000 bp product is obtained. In *osr1* morphants, intron 2 fails to be spliced out the gene, leading to a 631 bp increase in product (*). Further, the intronic sequence retained in *osr1* morphants contains in-frame stop codons. (**C**) *osr1* morphants had dramatically decreased podocytes and a cropped pronephric tubule which resembled both previously published results and *ocn-/-*. Scale bar is 30 µm.

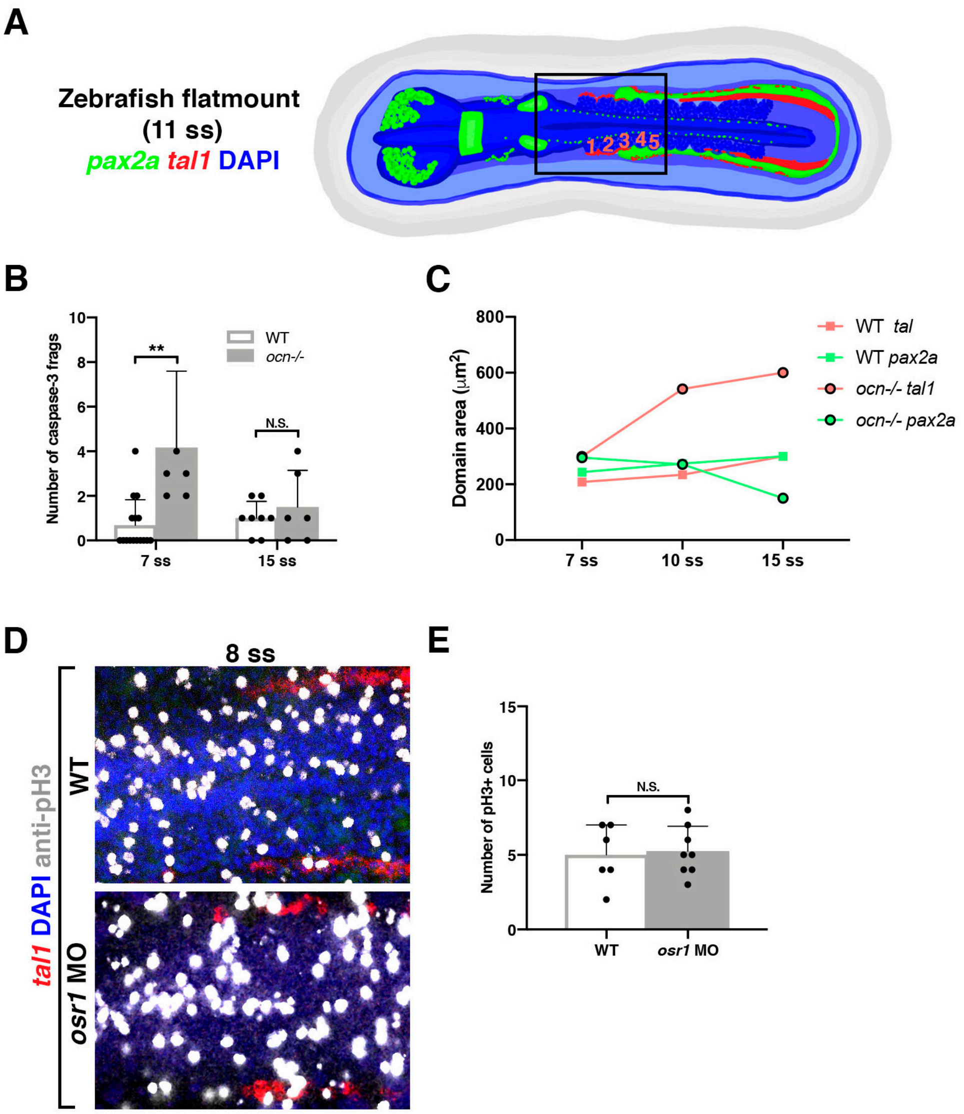

Supplemental Figure S4. Additional analysis of cell dynamics in *osr1* deficient models. **(A)** FISH experiments were conducted on young embryos (11 ss = 15 hpf) and flatmounted to be imaged. Probes for *pax2a* (green) were used to mark IM, and *tal1* (red), to mark hemangioblasts. DAPI (blue) marks nuclei and was used to distinguish cellular features such as muscular units known as somites. Counting somites allowed for accurate staging of embryos and a consistent location for tissue assessment. Areas and cell counts of mesodermal tissues were assessed from somites 1-5, as shown in the boxed area. (**B)** There was a significant increase in caspase-3+ fragments in the *tal1/pax2a* area of interest in *ocn-/-* compared to siblings at 7 ss. However, by 15 ss, the number of caspase-3+ fragments in *ocn-/-* had returned to a WT level. (**C)** Compared to WT siblings, the *tal1* domain steadily increased in *ocn-/-* from 7 to 15 ss. In contrast, the *pax2a* domain only became significantly smaller in *ocn-/-* mutants at the 15 ss. **(D,E)** There was no significant change in the number of pH3+ cells in *osr1* morphants in the *tal1* domain between somites 1-5 at the 8 ss. P-values: **p<0.001, and N.S. not significant.

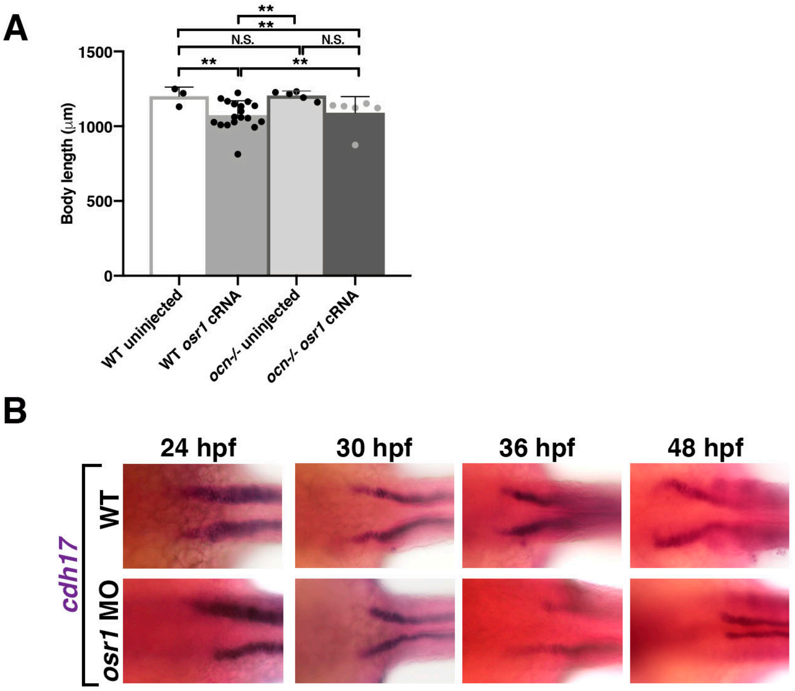

Supplemental Figure S5. *osr1* cRNA injection additional analysis. (**A**) *ocn-/-* and WT sibling embryos injected with *osr1* cRNA had significantly shorter body lengths than uninjected *ocn-/-* mutants and siblings. P-values: **p<0.001, N.S. = not significant. P-values were obtained by arcsin transforming percentages to normalize data. (**B)** Time course of *cdh17* expression in WT and *osr1* MO injected animals. Scale bar is 20 μm.

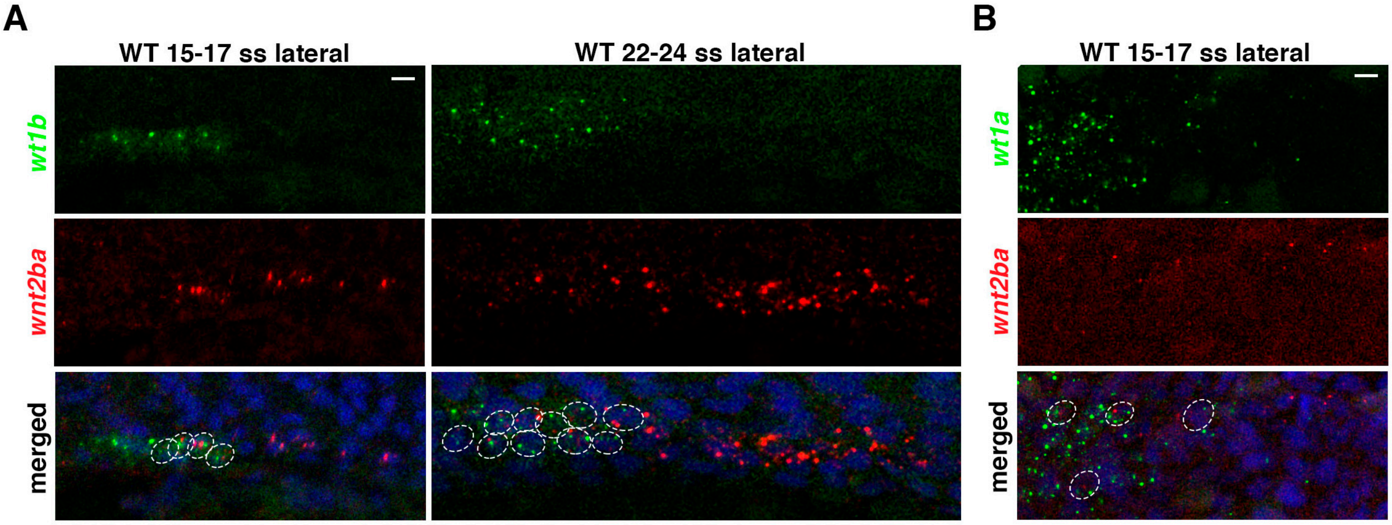

Supplemental Figure S6. *wnt2ba* colocalizes with early, developing podocytes (**A,B**) Lateral views of FISH experiments reveal that *wnt2ba* is present in developing *wt1a* and *wt1b* podocytes at 15-17 ss and 22-24 ss. Scale bar is 10 μm.

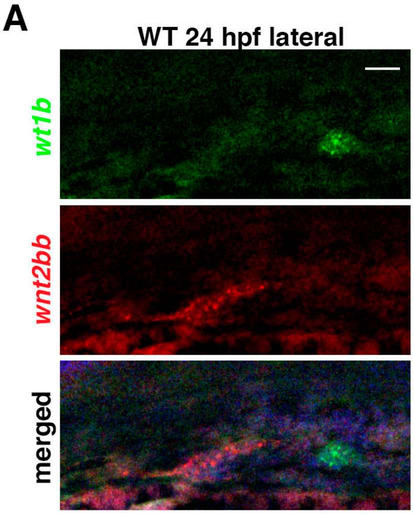

Supplemental Figure S7. *wnt2bb* does not colocalize with podocytes. **(A)** Lateral views of WT embryos stained using FISH reveal that *wnt2bb* is expressed anterior to *wt1b* podocytes at 24 hpf. Scale bar is 50 µm.

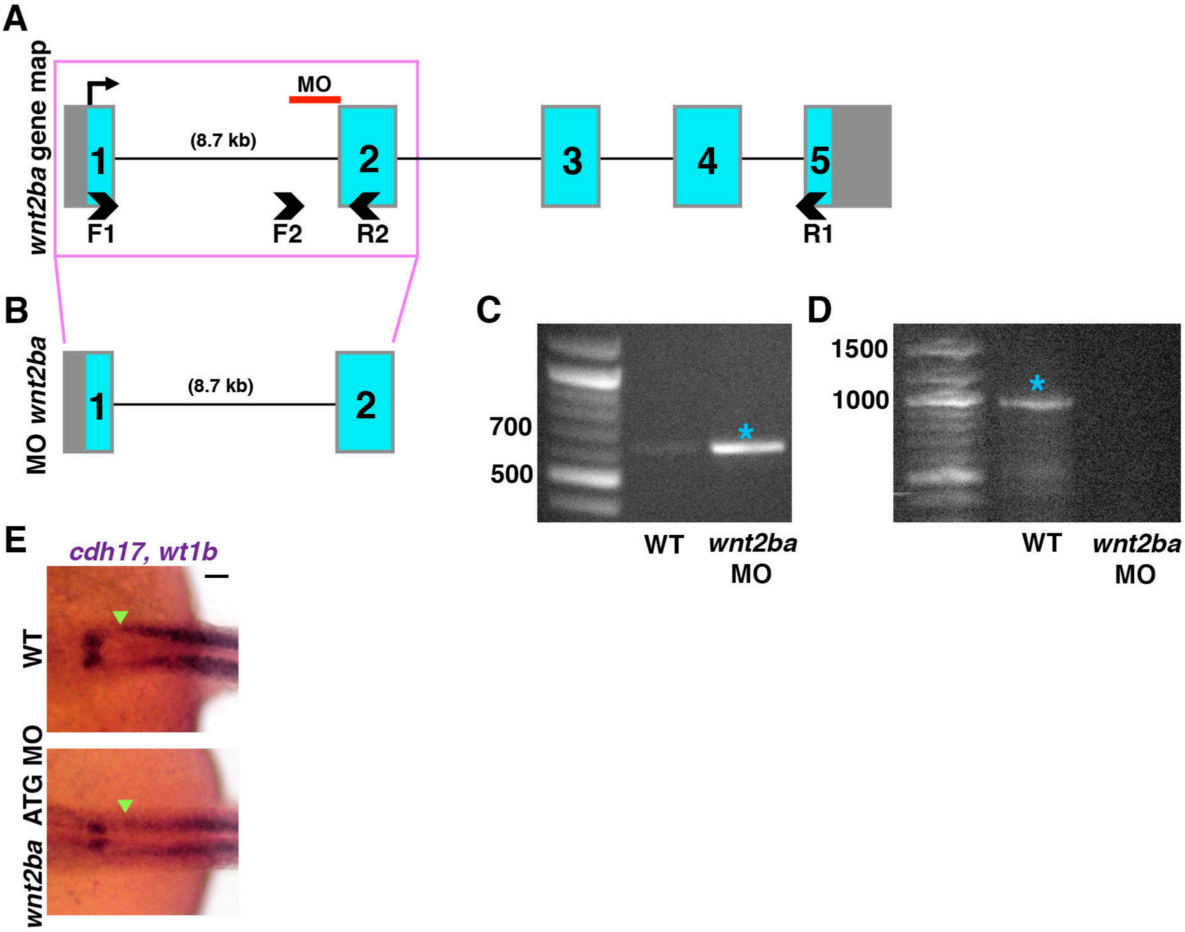

Supplemental Figure S8. Assessment of *wnt2ba* MO through RT-PCR analysis**. (A)** The gene *wnt2ba* contains 5 exons. A morpholino was utilized to block splicing activity in intron 1. **(B)** In WT embryos, correct splicing activity occurs to splice exon 1 and 2 together, resulting in a ~300 bp product as seen with F1/R1 primers. This product is greatly diminished in *wnt2ba* morphants, suggesting that correct splicing activity is limited. **(C)** Primers were utilized that flanked the end of intron 1 and the middle of exon 2 in *wnt2ba* (F2/R2). In *wnt2ba* morphants, a strong band (*) was present that indicated the presence of un-spliced, intronic sequence (**D)** Primers flanking the entire ORF (F1/R2) were used as an additional metric to gauge splicing action. WTs exhibit a 1000 bp band (*) which represents the *wnt2ba* ORF. *wnt2ba* morphants do not have this band, which further suggests that splicing activity is impaired. **(E)** An ATG MO was used to knockdown *wnt2ba*. While podocytes were reduced using this reagent, the pronephric tubule was not affected. The start of the tubule is shown using green arrowheads. Scale bar is 50 µm.

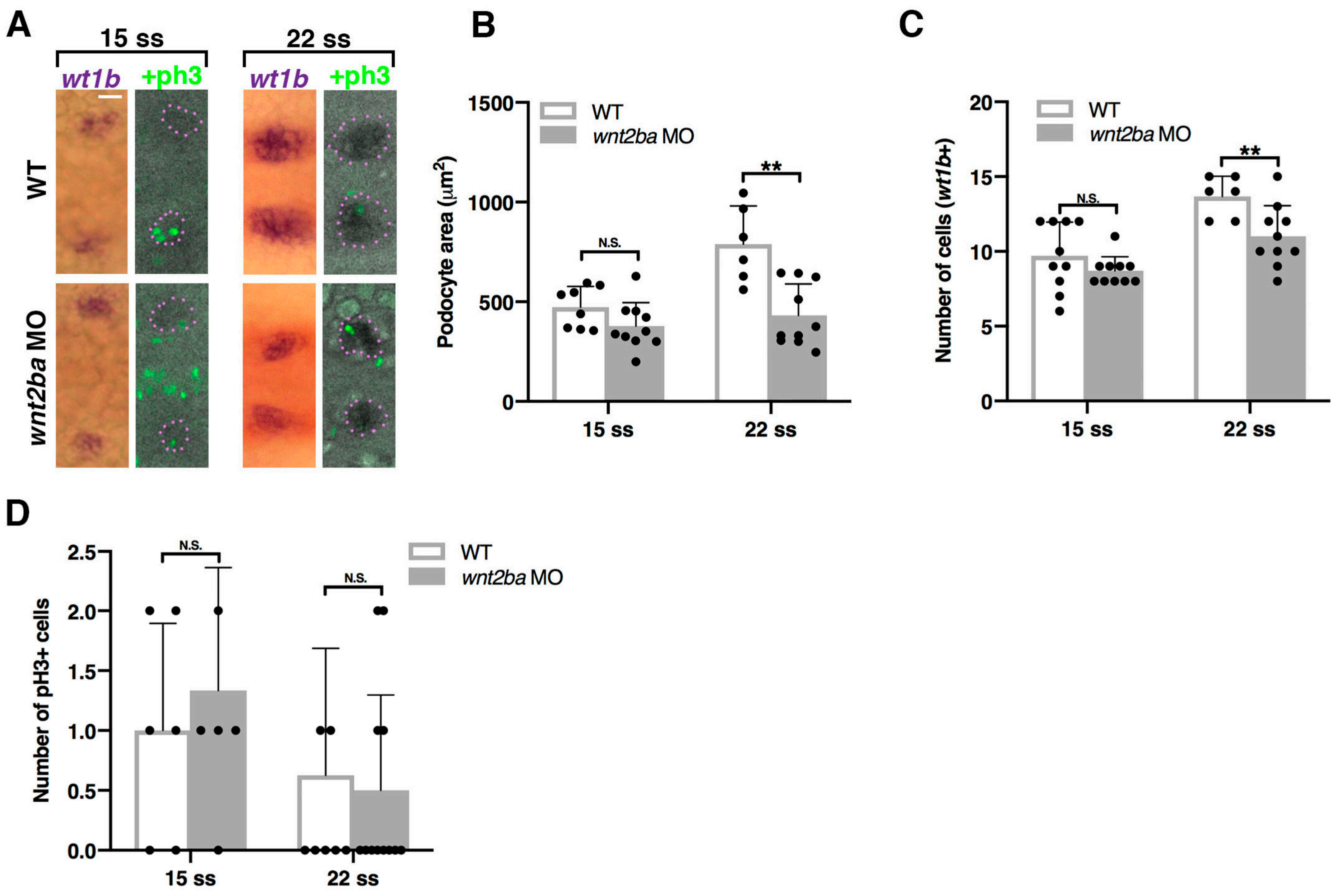

Supplemental Figure S9. *wnt2ba* knockdown causes decreased podocytes at 22 ss**.** (**A-D**) *wnt2ba* was knocked down using a splice-blocking MO and podocytes were examined by conducting WISH on 15 ss and 22 ss embryos using the marker *wt1b*. Anti-pH3 was used on these samples to assess proliferating cells. Podocyte area and cell counts were not different between morphants and WTs at 15 ss, though there was a significant decrease in podocyte cell number and domain area at 22 ss in morphants. However, there were no changes in the number of pH3+ cells in the *wt1b* area at either time point. Scale bar is 40 μm. P-values: **p<0.001, and N.S. not significant.

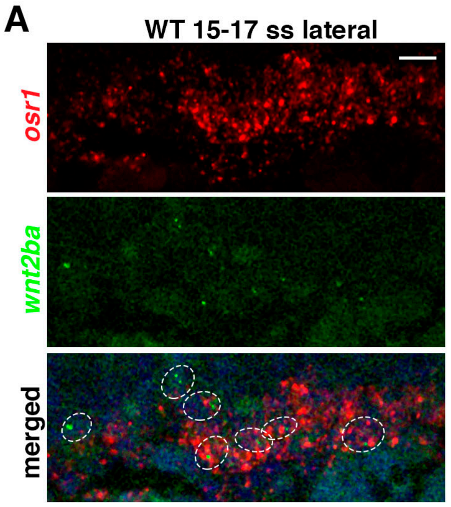

Supplemental Figure S10. Additional stages of *wnt2ba* and *osr1* co-localization**.** **(A)** In addition to 22 ss, it was also observed that *osr1* and *wnt2ba* colocalized in a subset of presumptive IM at 15-17 ss. Scale bar is 10 μm.

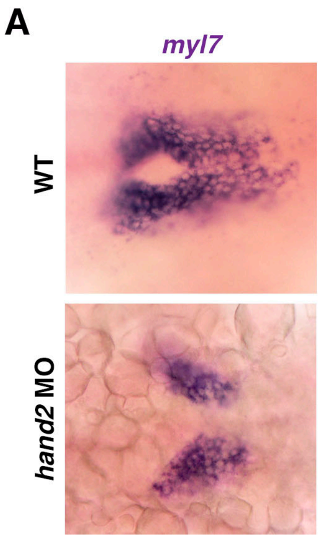

Supplemental Figure S11. *hand2* MO replicates previous studies. **(A)** The developing heart tube can be visualized with *myl7* at 22 ss. Embryos injected with *hand2* MO display cardia bifida, or a separation of heart precursors.
